# Null anticarcinogenic effect of silymarin on diethylnitrosamine-induced hepatocarcinogenesis in rats

**DOI:** 10.3892/etm.2013.1391

**Published:** 2013-11-07

**Authors:** RYU IMAMOTO, JUN-ICHI OKANO, SHINTARO SAWADA, YUKI FUJISE, RYO ABE, YOSHIKAZU MURAWAKI

**Affiliations:** Second Department of Internal Medicine, Tottori University School of Medicine, Yonago, Tottori 683-8504, Japan

**Keywords:** diethylnitrosamine, hepatocellular carcinoma, silymarin

## Abstract

The aim of this study was to investigate the anticarcinogenic effects of silymarin in diethylnitrosamine (DEN)-induced hepatocarcinogenic rat models. Severe and mild models of hepatocellular carcinoma (HCC) were generated by the intraperitoneal administration of 40 mg/kg DEN once a week for 18 weeks and 100 mg/kg DEN every 2 weeks for 6 weeks in male Wistar rats, respectively. In the severe and mild models of HCC, the rats were treated with 0.1 and 0.5% silymarin for 18 weeks and with 0.1% silymarin for 5 weeks, respectively. Serum transaminase levels were not significantly decreased by the silymarin treatment in either model. Macroscopic and microscopic features indicated that the silymarin-containing formulations did not significantly inhibit the hepatic tumor formation induced by DEN. Furthermore, immunohistochemical and western blot analyses demonstrated that the expression levels of proliferating cell nuclear antigen and glutathione *S*-transferase P, which are hepatocarcinogenic markers, were not significantly modified by the silymarin treatment. These results indicate that silymarin may not be considered as a candidate agent against hepatocarcinogenesis.

## Introduction

Hepatocellular carcinoma (HCC) is one of the most common malignancies in the world, ranking as the third most common cause of mortality from cancer (1,2) and the fifth most prevalent global malignancy (3). Since the majority of cases of HCC arise from livers diseased with chronic hepatitis and liver cirrhosis, caused by infection with hepatitis C virus (HCV) and hepatitis B virus (HBV), regular screening of these patients using ultrasonography (US) and tumor markers, including α-fetoprotein (AFP) and protein induced by vitamin K absence or antagonist-II (PIVKA-II), is required for the detection of HCC at an early stage (4). Despite advancements in the treatment of HCC and the early detection of HCC, the prognosis of patients with HCC remains unsatisfactory due to a high recurrence rate following treatment (5). In order to improve the outcomes of patients with chronic liver diseases, novel chemopreventive and therapeutic compounds that may be utilized for patients at a high risk of HCC and that exhibit no systemic toxicity are required.

Silymarin is a polyphenolic mixture of flavonoligands derived from the seeds of the milk thistle plant (*Silybum marianum*). Silymarin has been clinically applied for the treatment of liver diseases as an encapsulated, standardized extract, since it is not water-soluble (6). Silymarin has been indicated to possess hepatoprotective (7,8) and antihepatocarcinogenic (9–11) properties. The mechanisms of the antihepatocarcinogenic effects induced by silymarin include the inhibition of cell proliferation and the stimulation of apoptosis (12).

Diethylnitrosamine (DEN), which is present in tobacco smoke, cured and fried meals, cosmetics and pharmaceutical agents, has been established to be a powerful hepatocarcinogen in rats (13). The proposed mechanisms of DEN-induced hepatocarcinogenesis include the alteration of DNA structure, the formation of alkyl DNA adducts and the induction of chromosomal aberrations and micronuclei in the liver (14,15). In addition to a single injection of DEN followed by partial hepatectomy and coupled with 2-acetyl-aminofluorene (2-AAF) (16), the sequential administration of DEN for several weeks has been demonstrated to induce HCC in rodents (17,18). In the present study, severe and mild models of HCC were generated by the intraperitoneal administration of 40 mg/kg DEN once a week for 18 weeks and 100 mg/kg DEN every 2 weeks for 6 weeks in male Wistar rats, respectively. By establishing these DEN-induced rat models, the aim of the study was to evaluate the antihepatocarcinogenic effects of silymarin, with the aim of enabling the future administration of silymarin to patients with chronic liver diseases who are at high risk of HCC.

## Materials and methods

### Chemicals

Silymarin, DEN and an anti-β-actin antibody were purchased from Sigma Aldrich (St. Louis, MO, USA) and pentobarbital was obtained from Dainippon Sumitomo Pharma Co., Ltd. (Osaka, Japan). Antibodies against proliferating cell nuclear antigen (PCNA) and glutathione *S*-transferase (GST) P were purchased from Santa Cruz Biotechnology Inc. (Santa Cruz, CA, USA) and Assay Designs, Inc. (Ann Arbor, MI, USA), respectively. Secondary anti-mouse and anti-rabbit horseradish peroxidase (HRP) antibodies for western blot analysis were obtained from GE Healthcare Ltd. (Buckinghamshire, UK). All other chemicals and solvents used in this study were of analytical grade.

### Animals, treatments and tissue collection

Male Wistar rats (weight, ~200 g) were obtained from Japan SLC, Inc. (Hamamatsu, Japan). The rats were housed two per cage with rice husks for bedding in an air-ventilated room under a 12-h light/dark cycle. The temperature (22ºC) and humidity (55%) were kept constant. The animals were allowed free access to food and tap water *ad libitum* during the experiment. All animals received humane care and protocols were approved by the Animal Ethics Committee of Tottori University (Yonago, Japan). Two models were employed to evaluate the antihepatocarcinogenic effects of silymarin.

**Figure 1:**
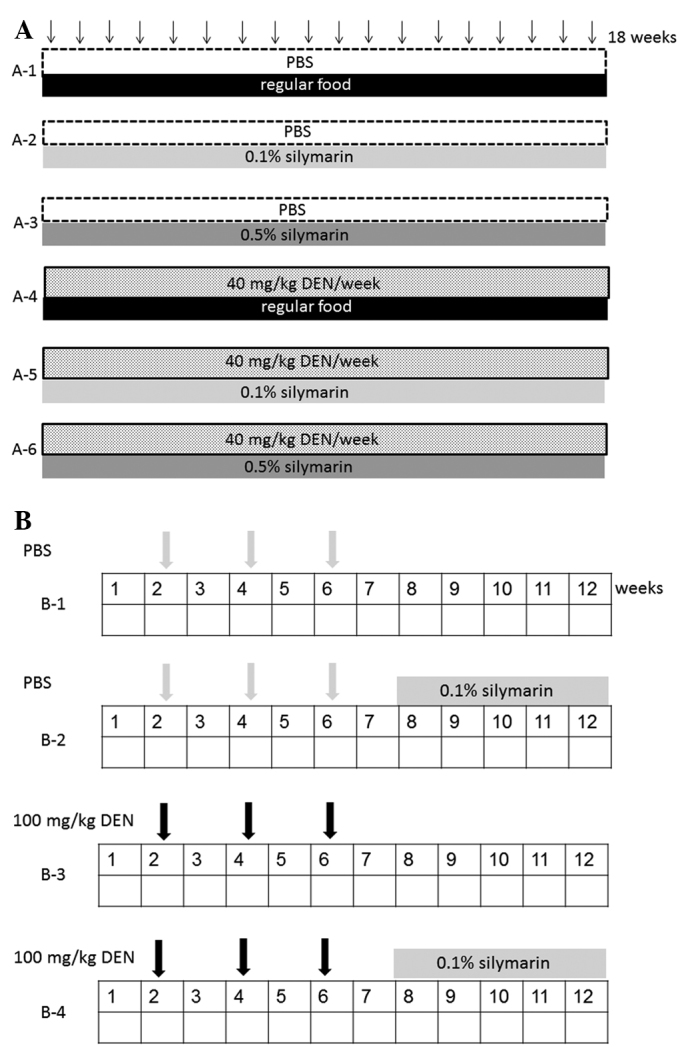
Experimental schedules. (A) In the severe hepatocellular carcinoma (HCC) model (model A), male Wistar rats were divided into six groups. The animals were intraperitoneally injected with 300 μl phosphate-buffered saline (PBS; groups A-1, A-2 and A-3; n=4) or diethylnitrosamine (DEN; 40 mg/kg body weight) dissolved in PBS weekly for 18 weeks (groups A-4, A-5 and A-6; n=4). The rats were fed with 0.1% silymarin (groups A-2 and A-5) and 0.5% silymarin (groups A-3 and A-6) in powder form for 18 weeks. One week subsequent to the 18-week treatments, the animals were sacrificed.(B) In the mild HCC model (model B), male Wistar rats were randomized and divided into four groups. The animals were intraperitoneally injected with 300 μl PBS (groups B-1 and B-2; n=8) or DEN (100 mg/kg body weight) dissolved in PBS (groups B-3 and B-4; n=8) at 15-day intervals on experimental weeks 2, 4 and 6. In groups B-2 and B-4, the rats were fed with 0.1% silymarin in powder form during experimental weeks 8 to 12. One week subsequent to the final treatments, the animals were sacrificed.

The study utilized severe (model A) and mild (model B) models of HCC, in which the animals were randomized and divided into six (Fig. 1A) and four (Fig. 1B) groups, respectively. In model A, the animals were intraperitoneally injected with 300 μl phosphate-buffered saline (PBS) (groups A-1, A-2 and A-3; n=4) or DEN (40 mg/kg body weight) dissolved in PBS weekly for 18 weeks (groups A-4, A-5 and A-6; n=4). In order to examine the preventive effects of silymarin on hepatocarcinogenesis, the rats were fed with 0.1% silymarin (groups A-2 and A-5) or 0.5% silymarin (groups A-3 and A-6) in powder form for 18 weeks. One week subsequent to the 18-week treatments, animals were sacrificed by cardiac puncture under anesthesia using pentobarbital.

In model B, the animals were intraperitoneally injected with 300 μl PBS (groups B-1 and B-2; n=8) or DEN (100 mg/kg body weight) dissolved in PBS (groups B-3 and B-4; n=8) once every 2 weeks on experimental weeks 2, 4 and 6. In groups B-2 and B-4, the rats were fed with 0.1% silymarin in powder form during experimental weeks 8 to 12 to examine the therapeutic effects of silymarin on the DEN-induced hepatocarcinogenesis. One week subsequent to the final treatments, the animals were sacrificed under anesthesia using pentobarbital. Blood samples were obtained via cardiac puncture and serum samples were stored at −30ºC until analysis. Immediately following the excision of the livers, the livers were divided into two sections for histological examination in 10% neutral buffered formalin and for protein studies at −80ºC.

### Measurement of serum transaminase levels

Serum aspartate aminotransferase (AST), alanine aminotransferase (ALT) and alkaline phosphatase (ALP) levels were measured at SRL, Inc. (Tokyo, Japan).

### Total protein preparation and western blotting

The liver samples were homogenized using a BioMasher^®^ (Nippi Inc., Tokyo, Japan) and lysed in radioimmunoprecipitation (RIPA) buffer (Millipore Corp., Bedford, MA, USA) supplemented with 1 mM sodium orthovanadate, 1 mM phenylmethylsulfonyl fluoride (PMSF) and a protease inhibitor mixture tablet (Roche Diagnostics, Basel, Switzerland) for 10 min on ice. Total protein samples (5 μg) were separated using sodium lauryl sulfate (SDS)-polyacrylamide gel electrophoresis (PAGE; SuperSep; Wako Pure Chemical Industries, Ltd., Osaka, Japan) and transferred to a polyvinylidene difluoride (PVDF) membrane (Immobilon-P; Millipore Corp.). Subsequent to the membranes being blocked in 5% non-fat milk (Santa Cruz Biotechnology Inc.) in 10 mM Tris, 150 mM NaCl (pH 8.0) and 0.1% Tween 20 (TBST) for 1 h at room temperature, they were probed with primary antibodies overnight at 4ºC, washed three times in TBST and incubated with anti-mouse or anti-rabbit HRP antibody in TBST for 1 h at room temperature. Following this, the signals were developed with a chemiluminescence solution (ECL; GE Healthcare Ltd.), visualized and quantified using an image analyzer (LAS-3000 mini; Fujifilm Co., Tokyo, Japan).

### Histology and immunohistochemistry

The rat liver tissues were fixed in 10% neutral buffered formalin and paraffin-embedded. For the histological analysis, serial sections (5-μm) were stained with hematoxylin and eosin (H&E). Neoplastic nodules and HCC were classified on the basis of the published criteria (19). For immunohistochemistry with the PCNA and GST-P antibodies, Histofine^®^ Simple Stain Rat MAX PO (Nichirei Biosciences Inc., Tokyo, Japan) was employed. Briefly, following routine dewaxing with xylene and hydration through a graded ethanol series, the sections were incubated with 1.5% hydrogen peroxide solution for 15 min at room temperature to terminate the endogenous peroxidase activity. The sections were subsequently washed in PBS, blocked with 1.5% serum solution and incubated with primary antibodies overnight at 4ºC. Following this, the sections were rinsed with PBS and incubated with biotinylated secondary antibody for 30 min at room temperature. In addition, HRP-conjugated avidin biotin complex (ABC) solution (Vector Laboratories, Inc., Burlingame, CA, USA) was applied for 30 min at room temperature. The peroxidase activity was developed using 3,3′-diaminobenzidine (DAB) solution (Vector Laboratories, Inc.). Counterstaining was performed using hematoxylin. The PCNA labeling indices were represented as the percentage of positively stained nuclei by counting 1,000 cells in a field at ×200 magnification. The GST-P-positive area was measured on images captured by a Charge Coupled Device (CCD) camera on a Windows^®^ computer.

### Statistical analysis

Values are expressed as the mean ± standard deviation. Values between two groups were compared using the Mann-Whitney U-test. P<0.05 was considered to indicate a statistically significant difference.

## Results

### Relative liver weight and serum transaminase levels

**Figure 2:**
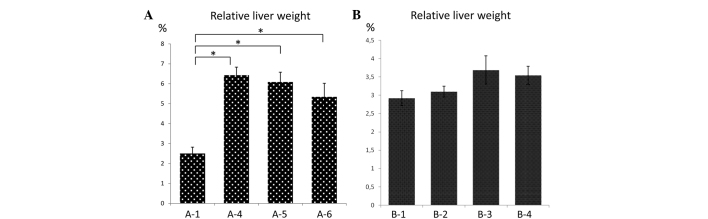
Relative liver weight (liver weight/body weight). Means of relative liver weight in (A) severe model groups A-1, A-4, A-5 and A-6 and (B) mild model groups B-1, B-2, B-3 and B-4. ^*^P<0.05.

**Figure 3:**
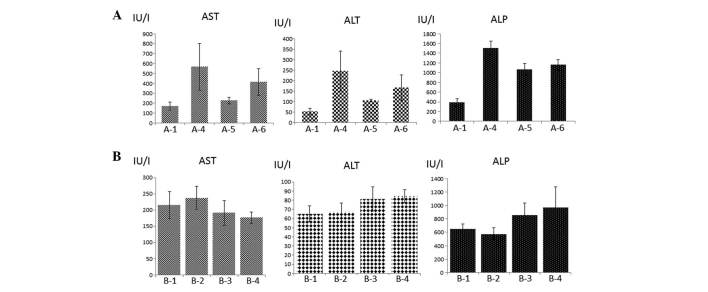
Serum transaminase and alkaline phosphatase (ALP) levels. Means of serum alanine aminotransferase (ALT), aspartate aminotransferase (AST) and ALP levels in groups (A) A-1, A-4, A-5 and A-6 and (B) B-1, B-2, B-3 and B-4.

In model A, two rats in group A-4 died during the experimental period. The relative liver weight (liver weight/body weight) was significantly higher in the DEN groups (A-4, A-5 and A-6) than in A-1, which was presumably due to the development of liver tumors. No significant differences were identified in relative liver weight among groups A-4, A-5 and A-6, irrespective of the silymarin treatment (Fig. 2A). Serum transaminase (AST and ALT) and ALP levels were higher in the DEN groups (A-4, A-5 and A-6) than in A-1, which most likely reflected the hepatic injury induced by DEN (Fig. 3A). No significant differences were identified in serum transaminase and ALP levels among groups A-4, A-5 and A-6. Relative liver weight and serum transaminase and ALP levels in groups A-2 and A-3 were not significantly different compared with the values in group A-1 (data not shown). In model B, one rat in group B-4 died during the experimental period. No significant differences were identified in the relative liver weight or serum transaminase and ALP levels among the four groups, B-1, B-2, B-3 and B-4 (Fig. 2B and 3B).

### Macroscopic and histological examinations

**Figure 4:**
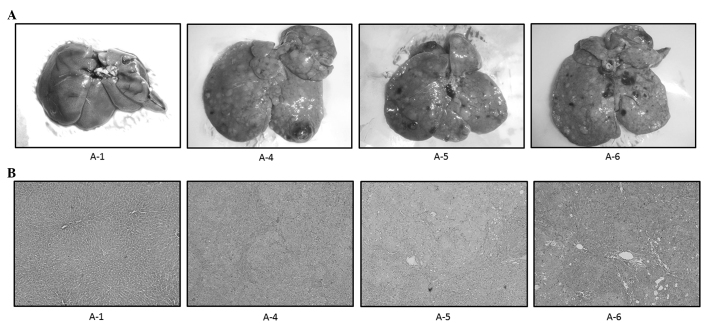
Representative (A) macroscopic and (B) microscopic features of the livers of model A rats. (A) In control rats treated with phosphate-buffered saline (PBS) without diethylnitrosamine (DEN), no tumors were observed (A-1, A-2 and A-3; data not shown for A-2 and A-3). Multiple white nodules were macroscopically observed following DEN administration, irrespective of silymarin treatment (A-4, A-5 and A-6). (B) In control rats treated with PBS without DEN, the liver histology showed a normal appearance (A-1, A-2 and A-3; data not shown for A-2 and A-3). In the histological analysis, white nodules induced by DEN were demonstrated to be hepatocellular carcinoma (HCC; A-4). The HCC area was not significantly modified by silymarin treatment (A-5 and A-6); original magnification, ×100.

**Figure 5:**
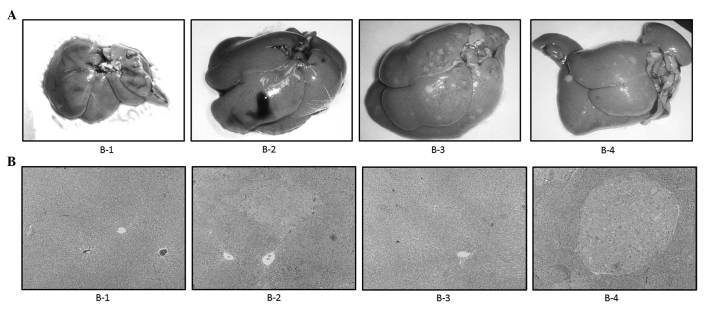
Representative (A) macroscopic and (B) microscopic features of the livers of model B rats. (A) Several white nodules were macroscopically observed following diethylnitrosamine (DEN) treatment (B-3). The gross appearance of the liver treated with DEN and 0.1% silymarin was mostly identical to that of the liver treated with DEN alone (B-4). (B) In the histological analysis, hyperplastic nodules were developed following treatment with DEN (B-3), which was not significantly modified by the silymarin treatment (B-4); original magnification, ×100.

Macroscopic and microscopic features of the liver were evaluated in the two models. As expected, in control rats without DEN treatment (A-1, A-2, A-3, B-1 and B-2), no tumors were observed (Figs. 4A and 5A for A1 and B1, respectively; data not shown for A-2, A-3 and B-2) and the liver histology showed a normal appearance (Figs. 4B and 5B for A1 and B1, respectively; data not shown for A-2, A-3 and B-2). In model A, multiple white nodules were macroscopically observed in the groups treated with DEN (Fig. 4A, group A-4). The gross appearance of the livers treated with DEN and 0.1 and 0.5% silymarin was predominantly identical to that of the liver treated with DEN alone, with no significant differences in the number of nodules among groups A-4, A-5 and A-6 (Fig. 4A). In the histological analysis, the white nodules were demonstrated to be HCC. Consistent with the macroscopic findings, the HCC area was not significantly modified by silymarin treatment (Fig. 4B).

In model B, a number of white nodules, although fewer than in model A, were macroscopically observed in the groups treated with DEN (Fig. 5A, group B-3). In the histological analysis, hyperplastic nodules were shown to have developed following DEN treatment (Fig. 5B, group B-3). The gross appearance of the liver treated with DEN and 0.1% silymarin was predominantly identical to that of the liver treated with DEN alone, with no significant difference in the number of nodules between B-3 and B-4 (Fig. 5B). Consistent with the macroscopic findings, the nodular area was not significantly modified by the silymarin treatment (Fig. 5B). These results indicated that silymarin did not have a significant impact on hepatitis or hepatocarcinogenesis induced by DEN in the severe or mild model of hepatocarcinogenesis.

### Expression levels of PCNA and GST P

**Figure 6:**
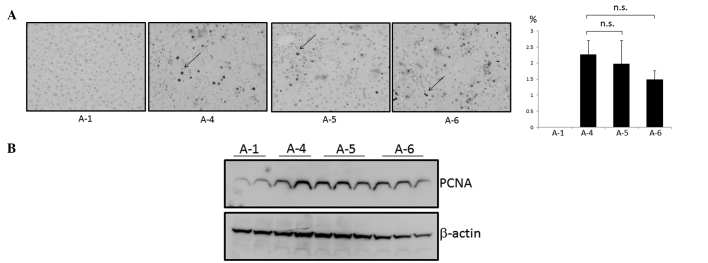
Expression of proliferating cell nuclear antigen (PCNA) in the livers of model A rats. Expression levels were evaluated using (A) immunohistochemical and (B) western blot analyses. (A) Representative liver tissues immunostained with anti-PCNA antibody in the control (A-1), diethylnitrosamine (DEN; A-4), DEN with 0.1% silymarin (A-5) and DEN with 0.5% silymarin (A-6) groups (top left panel). Arrows indicate the representative PCNA-positive cells. Percentages of PCNA-positive cells in A-1, A-4, A-5 and A-6 were 0, 2.3, 2.0 and 1.5%, respectively (top right panel); original magnification, ×400. (B) Representative liver samples from groups A-1, A-4, A-5 and A-6 were probed with anti-PCNA antibody (top lane). The membrane was reprobed with anti-β-actin antibody (bottom lane). n.s., not significant.

**Figure 7:**
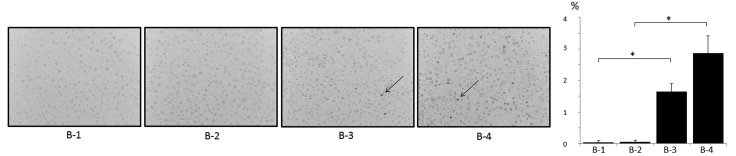
Expression of proliferating cell nuclear antigenin (PCNA) in the livers of model B rats. Representative liver tissues immunostained with anti-PCNA antibody in the control (B-1), 0.1% silymarin (B-2), diethylnitrosamine (DEN; B-3) and DEN with 0.1% silymarin (B-4) groups (left panel). Arrows indicate the representative PCNA-positive cells. Percentages of PCNA-positive cells in B-1, B-2, B-3 and B-4 were 0, 0, 1.7 and 2.9%, respectively (right panel); original magnification, ×400. ^*^P<0.05.

PCNA is an essential regulator of the cell cycle and its expression is a useful tool for the study of cell proliferation, including in the liver (20). The expression levels of PCNA in the liver among the treatment groups of models A and B were examined. Immunohistochemical analysis revealed that PCNA-positive cells were scarcely observed in the control livers without DEN treatment (A-1, A-2, A-3, B-1 and B-2; Figs. 6A and 7 and data not shown). Following treatment with DEN, the number of PCNA-positive cells was significantly increased in the two models (Figs. 6A and 7). The number of PCNA-positive cells was not significantly altered following treatment with silymarin in model A (Fig. 6A), which was demonstrated using western blot analysis (Fig. 6B). However, treatment with silymarin in model B appeared to increase the number of PCNA-positive cells; the mechanisms for this are unknown (Fig. 7).

**Figure 8:**
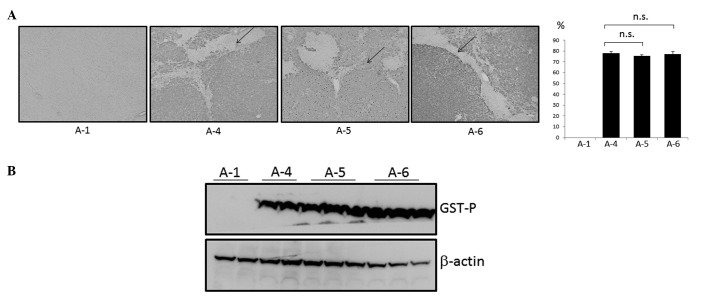
Expression of glutathione *S*-transferase P (GST P) in the livers of model A rats. Expression levels were evaluated using (A) immunohistochemical and (B) western blot analyses. (A) Representative liver tissues immunostained with anti-GST P antibody in the control (A-1), diethylnitrosamine (DEN; A-4), DEN with 0.1% silymarin (A-5) and DEN with 0.5% silymarin (A-6) groups (top left panel). Arrows indicate the representative GST P-positive area. Percentages of GST P-positive areas in A-1, A-4, A-5 and A-6 were 0, 78.2, 75.5 and 77.4%, respectively (top right panel); original magnification, ×100. (B) Representative liver samples from groups A-1, A-4, A-5 and A-6 were probed with anti-GST P antibody (top lane). The membrane was reprobed with anti-β-actin antibody (bottom lane). n.s., not significant.

**Figure 9:**
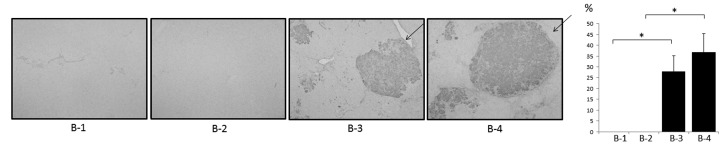
Expression of glutathione *S*-transferase P (GST P) in the livers of model B rats. Representative liver tissues immunostained with anti-GST P antibody in the control (B-1), 0.1% silymarin (B-2), diethylnitrosamine (DEN; B-3) and DEN with 0.1% silymarin (B-4) groups (left panel). Arrows indicate the representative GST P-positive area. Percentages of GST P-positive areas in B-1, B-2, B-3 and B-4 were 0, 0, 28.0 and 36.8%, respectively (right panel); original magnification, ×100. ^*^P<0.05.

Among the glutathione *S*-transferases (GSTs), a family of detoxification enzymes catalyzing the conjugation of glutathione with a large number of carcinogens, placental GST (GST P) is specifically expressed during rat hepatocarcinogenesis and has been used as a reliable tumor marker for experimental hepatocarcinogenesis in rats (21). As a result of this, the expression levels of GST P in the livers of the rats in the model A and B treatment groups were examined. Immunohistochemical and western blot analyses revealed that a GST P-positive area appeared in the DEN-treated livers (Figs. 8 and 9). However, expression levels of GST P were not significantly modified by the treatment with silymarin at any condition (Figs. 8 and 9). The combined results indicate that silymarin is not a potent compound useful for either the prevention or treatment of HCC.

## Discussion

Based on the fact that the majority of cases of HCC are complicated by chronic liver diseases, including chronic hepatitis and liver cirrhosis associated with HBV and HCV infection, the regular screening of patients who are at high risk of HCC, using US and computed tomography (CT), has been proposed in Japan to enable the early detection of HCC (4). However, HCC is often diagnosed at an advanced stage due to the inefficiency of US instrument operators, dropouts among the patients targeted by the screening program and an increasing incidence of non-B non-C HCC, which is difficult to include in the screening program (22,23). In addition to these complications hindering the early diagnosis of HCC, sorafenib, a multi-kinase inhibitor, has been demonstrated to exert marginally beneficial effects on the survival of patients with advanced HCC (24). Since the prognosis remains poor for patients with HCC, particularly at advanced stages, novel preventive and therapeutic approaches for these patients are urgently required.

Cancer chemoprevention is defined as ‘the use of specific natural or synthetic chemical agents to reverse or suppress carcinogenesis and prevent the development of invasive cancer using physiological pathways’ (25). Phytochemicals, which are plant-derived chemicals contained in fruits, vegetables and grains, have been subject to investigation due to their apparent antitumor activity against a variety of cancers, including HCC (26). Possible compounds with a potential to exert chemopreventive effects on the liver include caffeine, capsaicin, cinnamaldehyde, curcumin, epigallocatechin-3-gallate (EGCG), resveratrol, sulforaphane (SFN) and silymarin (27).

Among these phytochemicals, silymarin is a polyphenolic mixture of flavonoligands derived from the seeds of the milk thistle plant (*Silybum marianum*). Silymarin is a complex of five major compounds, silibinin, silychristin, isosilychristin, silydianin and taxifolin (8). Silymarin has been widely used as a natural remedy for the treatment of liver diseases since the time of the Ancient Greeks (12). The Hepatitis C Antiviral Long-Term Treatment against Cirrhosis (HALT-C) trial was designed to evaluate the efficacy of long-term treatment with low-dose peginterferon α-2a in patients with fibrosis and cirrhosis associated with hepatitis C, who had failed previous peginterferon and ribavirin therapy. Nearly one-third of the patients in the trial were former or current users of silymarin (28). Silymarin has been demonstrated to exhibit anticancer effects against cancers at a variety of sites, including the prostate (29), urinary bladder (30) and colon (31). Furthermore, silymarin has been indicated to display hepatoprotective (7,8) and antihepatocarcinogenic (9–11) properties in the liver.

In the present study, the antihepatocarcinogenic effects of silymarin in severe (model A) and mild (model B) models of HCC were explored. In model A, a low concentration of DEN (40 mg/kg body weight) was intraperitoneally administered weekly in a long-term course (18 weeks), which generated multiple HCCs in the rats. Treatment with silymarin was initiated from the beginning of DEN administration, in order to investigate the preventive effect of silymarin on HCC. In model B, a high concentration of DEN (100 mg/kg body weight) was intraperitoneally administered once every 2 weeks and silymarin was administered during experimental weeks 8 to 12, in order to explore the therapeutic effects of silymarin on the DEN-induced hepatocarcinogenesis. The doses of silymarin were selected to be 0.1 and 0.5% based on previous studies (12,32,33) and a preliminary experiment in which we tested the administration of 1% silymarin. Food intake and body weight gain with 1% silymarin were poor (data not shown). In the present study, under the selected experimental conditions, the results did not demonstrate the antihepatocarcinogenic effects of silymarin. Furthermore, silymarin appeared to accelerate the DEN-induced hepatocarcinogenesis (Fig. 7). There are several plausible explanations for these results, including: (i) the serum concentrations of silymarin attained were not high enough to exert biological effects, due to a low bioavailability of silymarin, although this was not measured; (ii) the hepatocarcinogenic models were too harsh to observe the antihepatocarcinogenic effects of silymarin; and (iii) silymarin does not possess antihepatocarcinogenic potential. Certain previous studies have revealed results consistent with the null anticarcinogenic effect of silymarin on DEN-induced hepatocarcinogenesis in rats that was observed in the present study (28,34). Silymarin potentiated ethanol-dependent HCC progression in mice (34), while users of silymarin had similar serum transaminase and HCV levels to those of nonusers in the HALT-C trial (28). In order to provide firm conclusions concerning the role of silymarin on hepatocarcinogenesis, further intensive investigations are required.

## Abbreviations

ALP: alkaline phosphatase
ALT: alanine aminotransferase
AST: aspartate aminotransferase
DEN: diethylnitrosamine
GST: glutathione *S*-transferase
H&E: hematoxylin and eosin
HBV: hepatitis B virus
HCC: hepatocellular carcinoma
HCV: hepatitis C virus
PCNA: proliferating cell nuclear antigen
